# EBR Strengthening Technique for Concrete, Long-Term Behaviour and Historical Survey †

**DOI:** 10.3390/polym10010077

**Published:** 2018-01-17

**Authors:** Christoph Czaderski, Urs Meier

**Affiliations:** Swiss Federal Laboratories for Materials Science and Technology (Empa), Überlandstrasse 129, 8600 Dübendorf, Switzerland; urs.meier@empa.ch

**Keywords:** externally bonded reinforcement, EBR, strengthening of reinforced concrete, epoxy adhesive, bond, steel, CFRP, long-term behaviour

## Abstract

Epoxy bonded steel plates (externally bonded reinforcemen: EBR) for the strengthening of concrete structures were introduced to the construction industry in the late 1960s, and the use of fibre reinforced polymers (FRPs) was introduced in the 1990s, which means that these techniques have already been used in construction for 50 and 25 years, respectively. In the first part of the paper, a historical survey of the development and introduction of these strengthening techniques into the construction industry are presented. The monitoring of such applications in construction is very important and gives more confidence to this strengthening technique. Therefore, in the second part of the paper, two long-term monitoring campaigns over an extraordinarily long duration will be presented. Firstly, a 47-year monitoring campaign on a concrete beam with an epoxy bonded steel plate and, secondly, a 20-year monitoring campaign on a road bridge with epoxy bonded CFRP (carbon fibre reinforced polymers) strips are described. The paper is an expanded version of the paper presented at the SMAR2017 Conference.

## 1. Historical Survey of Development of EBR Technique

### 1.1. Research on EBR Steel

**Figure 1:**
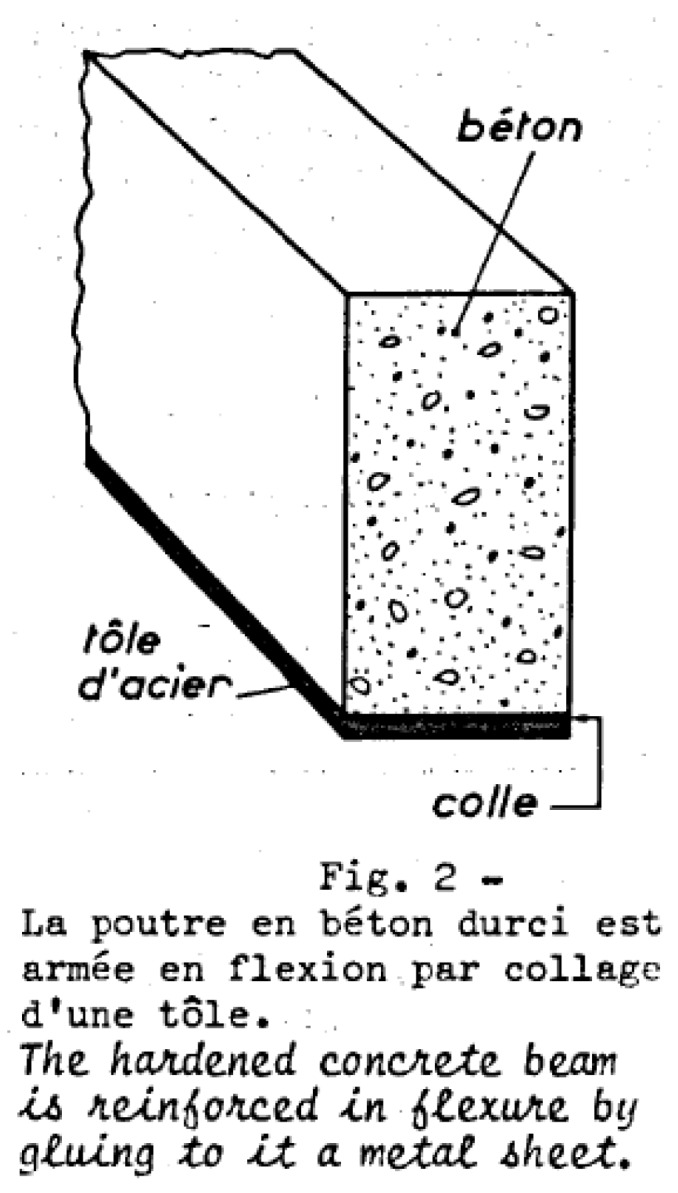
The idea of externally bonded reinforcement presented by Bresson [1] in the year 1967, taken from [1].

**Figure 2:**
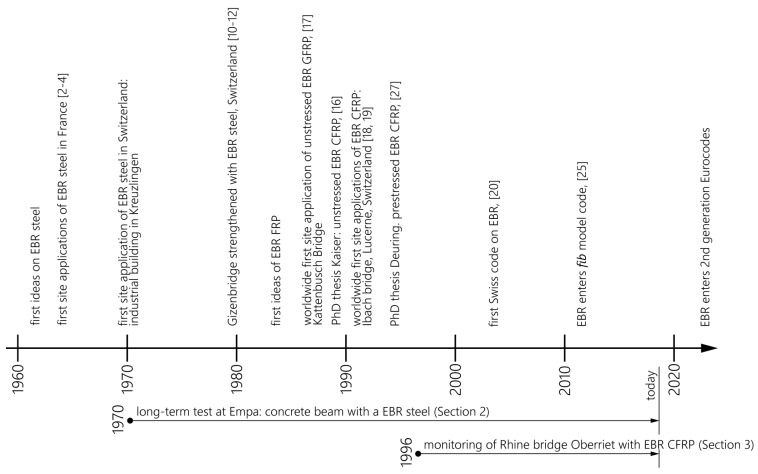
Timeline of the developments of the strengthening of reinforced concrete (RC) by externally bonded reinforcement (EBR).

The first ideas on strengthening of concrete with epoxy bonded steel plates were presented in the 1960s by L’Hermite et al. [1] and Bresson [2]. A figure taken from [1] is given in Figure 1 showing the principle idea of applying a steel plate to a concrete beam with an adhesive (tole d’acier: steel plate and colle: adhesive). The first applications on a highway bridge and in a building followed in 1966–1967 [3]. Bresson [2] presented in the year 1971 the derivation of the differential equation of bond behaviour of EBR and its elastic solution. Afterwards, other researchers worked on that topic—for example, Johnson et al. [4] presented in 1981 a research study on sixteen tests on plated reinforced concrete members subjected to bending, shear and axial tension, in order to study the influence of geometric parameters on the maximum tensile strain that can be developed in a mild steel plate before breakdown of the adhesive layer or failure of the concrete member. No failures occurred in the epoxy adhesive, and strains exceeding yield could be developed in the plates. Furthermore, Dussek [5] described results of tests on plate-reinforced beams, discussed a design method and presented strengthening projects where steel plates were applied on several bridges in UK. A timeline of the developments discussed is displayed in Figure 2.

**Figure 3:**
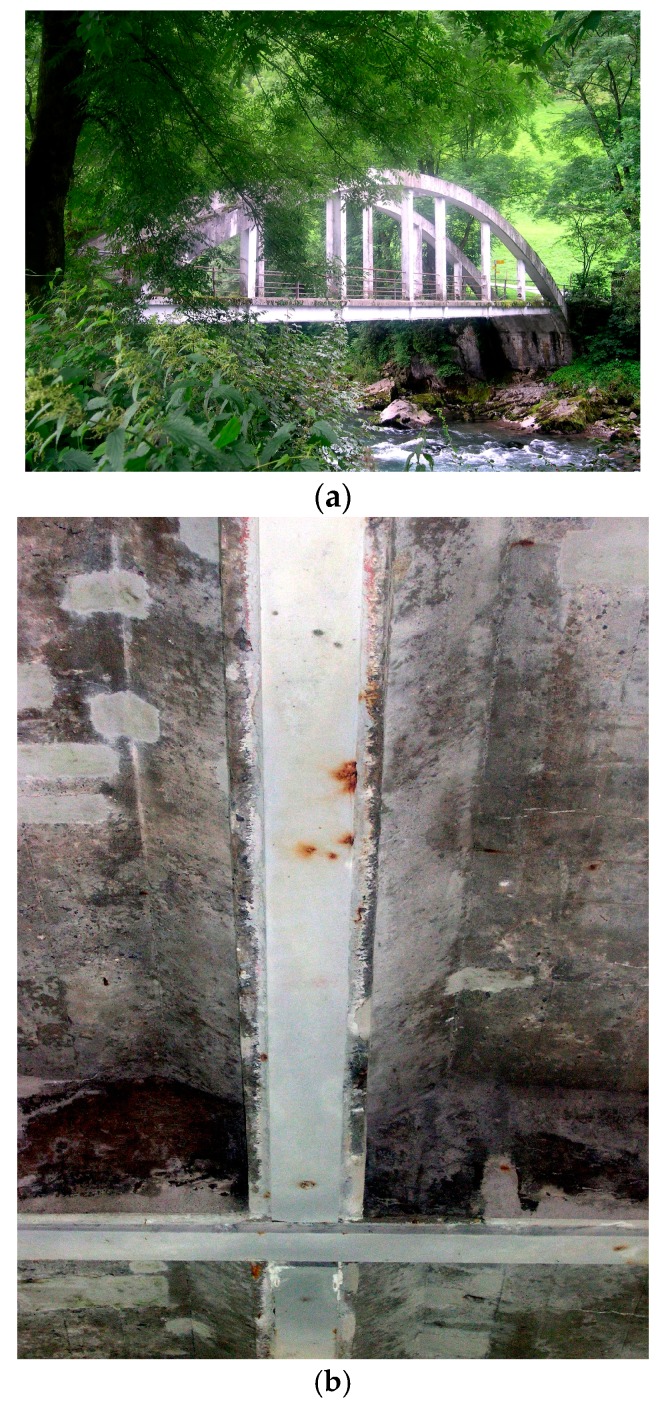
(**a**) Gizenenbridge in Switzerland and (**b**) bottom side of the bridge with steel plates (both photos in the year 2005).

In the 1970s and 1980s, besides the investigations mentioned above, research on the performance and on a better understanding on the epoxy bonded steel plates was also performed at Empa in City, Switzerland. An overview of these investigations at Empa is given in [6]. Bond tests, static and fatigue beam tests, long-term tests and completed strengthening projects were performed and are described in [6]. For a building in Zurich, Switzerland, which was strengthened by steel plates in April 1973, a large-scale test up to failure was performed [6,7]. Details of the strengthening work in the building are presented in Agthe [8]. One of the first bridges that was strengthened with epoxy bonded steel plates was the Gizenenbridge in Switzerland [9,10] in the year 1980. The bottom side of the bridge with the steel plates is visible on the photo given in Figure 3. Loading tests on the bridge after the strengthening are described in [11]. 

### 1.2. Research on EBR CFRP

**Figure 4:**
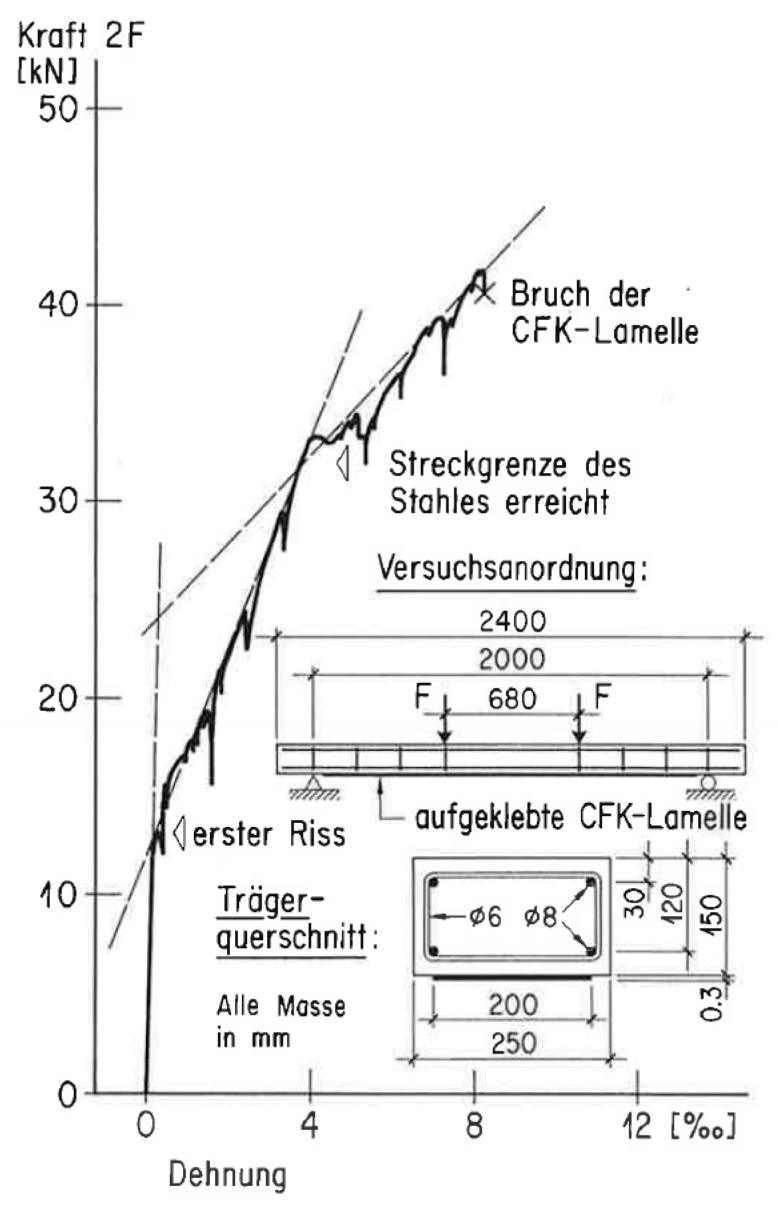
Typical load-displacement diagram of a CFRP strengthened RC beam, showing first cracking and yielding of the internal steel, published in German and French in the year 1987 [14]. Source Empa.

Carbon fibre reinforced polymers (CFRP) are used for load carrying components in the air and space industry since the 1970s. Empa researchers developed the idea of using CFRPs instead of steel in the 1980s. An analysis showed that the employment of advanced composites (e.g., carbon fibre reinforced epoxy laminates) reduced the costs for the scaffolding in the subsequent strengthening of bridges. Since reinforcement plates made of advanced composites were extremely light, scaffolding was not necessary [12]. Further advantages were the high stiffness, excellent fatigue properties and its outstanding corrosion resistance [13]. A typical load-displacement diagram of a CFRP strengthened RC beam was published already in 1987 in [14] and is shown in Figure 4. In the framework of a media event, Empa experts presented in 1987 that CFRP can also be used for strengthening of civil constructions [14]. The public interest was large; however, due to the high costs of the carbon fibres, the transfer to practice was first mistrusted. However, the handling of the very lightweight CFRP strips was much easier in comparison to steel plates and working hours could be saved, which compensated for the higher material costs of CFRP. The first PhD thesis on this topic was performed at Empa under supervision of the second author (1985–1989) [15].

**Figure 5:**
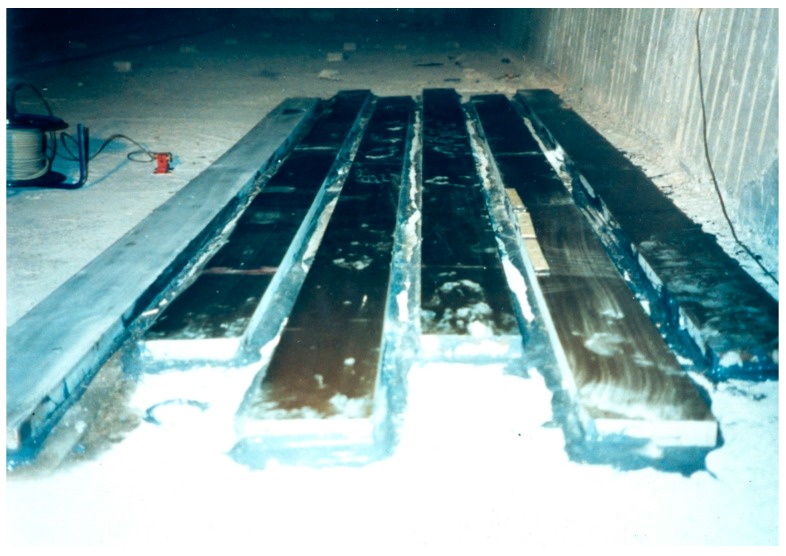
Glass fiber reinforced polymer (GFRP) Plates at the bottom slab of Kattenbusch Bridge (Source: Prof. Ferdinand S. Rostásy).

The Kattenbusch Bridge in Germany was the world’s first bridge where the EBR strengthening technique Glass Fibre Reinforced Polymers (GFRP) were applied [16]. It is a multispan box girder bridge built as an in situ spanwise construction. The working joints are at the points of contra flexure where the tendons are coupled. In the 1980s, cracks were observed at these working joints at the Kattenbusch Bridge. Due to increased fatigue stresses, the durability of the reinforcement and the tendons of the bottom slab were no longer assured. Prof. Ferdinand S. Rostásy recommended the building authorities the use of the EBR strengthening technique with GFRP plates. These plates were due to the low modulus of elasticity of GFRP relatively thick (Figure 5). The building authorities followed the recommendation and, to the best knowledge of the authors, these GFRP plates still fulfill their tasks. The application was in the year 1987.

**Figure 6:**
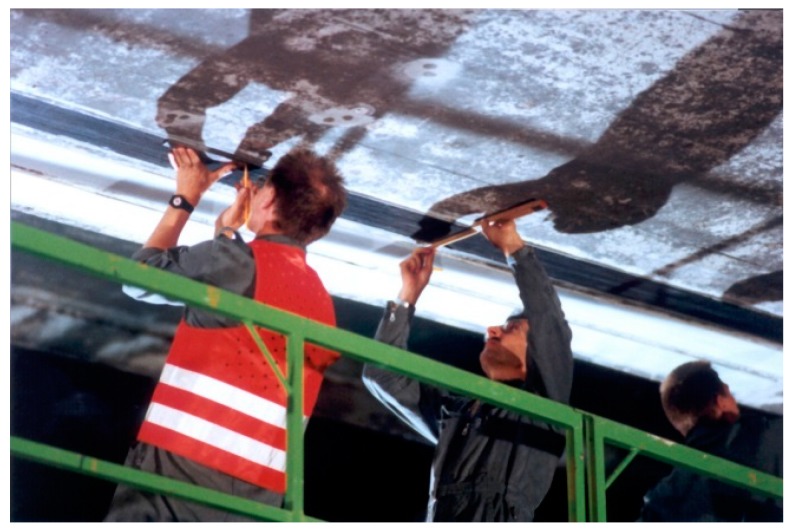
Strengthening work on the Ibach bridge in the year 1991.

**Figure 7:**
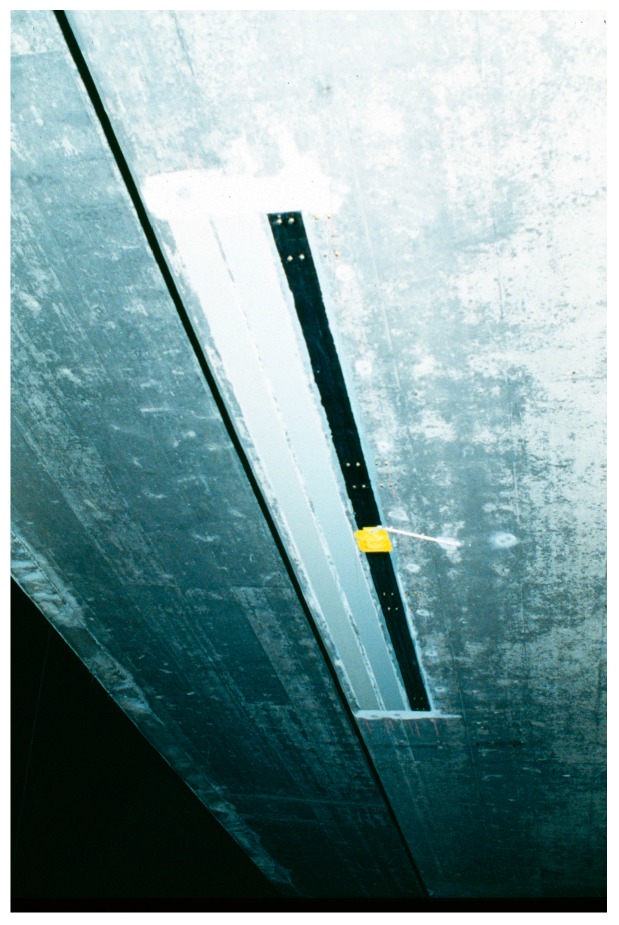
Applied CFRP strips on the Ibach bridge in the year 1991 (two strips were painted for aesthetic reasons).

**Figure 8:**
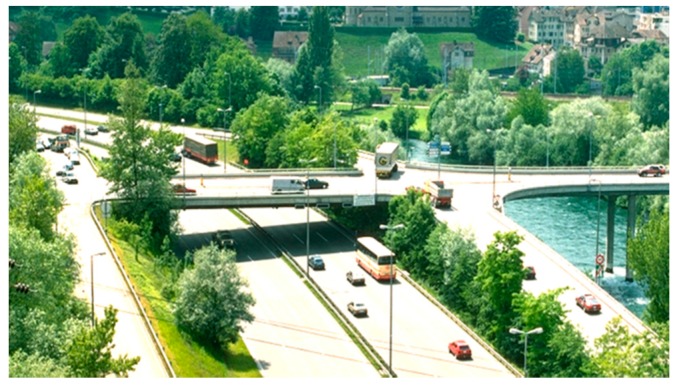
The Ibach Bridge near Lucerne, Switzerland.

As a world premiere, the Ibachbridge near Lucerne in Switzerland was successfully strengthened with CFRP strips in 1991 [17,18]. See Figure 6, Figure 7 and Figure 8. Core borings were performed to mount new traffic signals on the bridge and, in the process, a prestressing tendon in the outer web was accidentally damaged with several of its wires completely severed by means of an oxygen lance. The bridge was therefore repaired with three CFRP laminates; two had dimensions of 150 mm × 5000 mm × 1.75 mm and one had dimensions 150 mm × 5000 mm × 2 mm [18]. They had a fibre content of 55% (vol %) and an elastic modulus of 129 GPa. 

**Figure 9:**
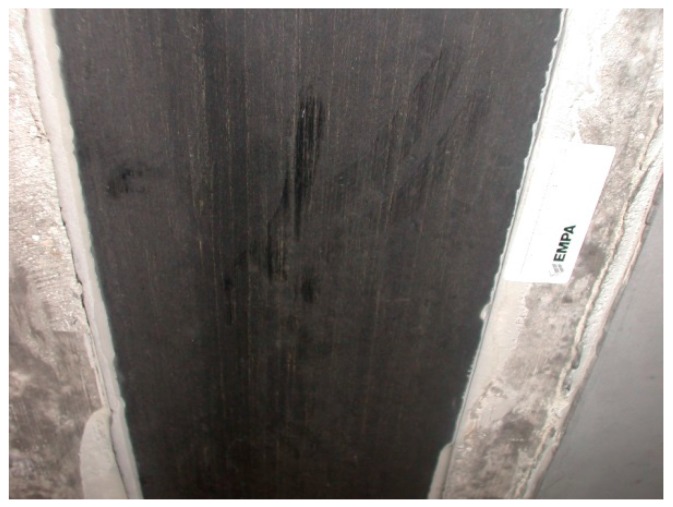
The photo shows the very good condition of one of the CFRP strips in the year 2008 (dust on the surface is visible).

Figure 9 shows a photo from the year 2008 of one of the CFRP strips, which were applied in 1991. It is visible that the CFRP strip was still in good condition, and only some dust is visible.

### 1.3. Research on Prestressed EBR CFRP

The idea of prestressing the CFRP laminates was firstly studied in a PhD thesis work by Deuring [19]. The prestressing of the CFRP strips has the advantages that the serviceability of the structure can be increased, i.e., the deformations and crack widths are decreased and the CFRP material is better utilized. Nowadays, several products are available on the marked to apply prestressed CFRP strips [20].

### 1.4. Transfer to Industry

For the transfer of new products and techniques to the industry, it is essential that guidelines exist. The first code in Switzerland on externally bonded reinforcement was published in 2004, [21], and also other countries released their guidelines in this field at that time, e.g., [22,23,24]. The bond of non-metallic reinforcement entered the *fib* model code 2010 [25,26] and work is going on in order to introduce them into the second generation of the Eurocodes.

**Figure 10:**
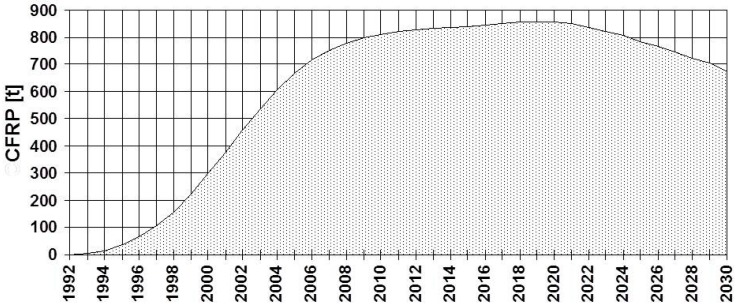
Taken from Meier [27]: Prediction of the worldwide demand of CFRP for external flexural post-strengthening in 1997. In 2014, the demand was in reality 7500 metric tons!

The FRP strengthening technique for reinforced concrete structures is nowadays state-of-the-art and is introduced into the industry. Several companies sell the FRP strips or fabrics and the suitable epoxy adhesive. The second author predicted in 1997 the worldwide demand of CFRP for EBR as given in Figure 10. Due to the lightweight, easy handling, good durability and high strength, the technique is extremely successful also from an economic point of view, which shows the number of companies that are working in the field. The prediction from 1997 was very much on the conservative side because the demand in 2014 was in reality 7500 tons.

### 1.5. Long-Term Behaviour

As described, this material is used already for 25 years in construction. However, the application of this material requires not only knowledge of the ultimate and serviceability limit states, but also information on the durability of the CFRP over the remaining lifetime of the structures. Many investigations on the durability and long-term behaviour of unstressed and prestressed externally bonded reinforcements are available in the literature. For example, Correia et al. [28] investigated the durability of RC slabs strengthened with prestressed CFRP laminate strips according to the EBR technique by exposing strengthened RC specimens for approximately eight months in water at 20 °C, in water with 3.5% dissolved chlorides at 20 °C and wet/dry cycles in a tank with a water temperature of 20 °C. It was found that the environmental conditions and the sustained loading, separately or combined, led in general to slight losses of performance and ductility. Similarly, Harmanci et al. [29] presented an experimental investigation on the long-term resistance of the gradient anchorage, a purely epoxy-based non-mechanical anchoring technique for prestressed carbon fiber reinforced polymer (CFRP) strips, after exposure to accelerated ageing conditions. Several exposure scenarios and their effect on the residual load carrying capacity were considered, namely the effect of carbonated concrete, freeze–thaw cycles, as well as their combination. Results indicated a higher anchorage resistance for carbonated concrete compared to the reference specimens. However, specimens subjected to freeze–thaw cycles exposure suffered from a significant reduction in residual anchorage resistance, as well as a shift in failure mode from a concrete substrate dominated to an epoxy/concrete interface failure.

Moreover, in a study by Kim et al. [30], carbon reinforced polymer and glass reinforced polymer materials were bonded to the tension face of two RC beams. The beams were then placed under sustained loads for 300 days. Displacements and strains were monitored. No failure occurred. The proposed method to predict the long-term deformations showed good agreement with the experimental results. Furthermore, it was concluded that, under the same sustained loads, specimens that were externally bonded with FRPs showed less time-dependant deflections than those of the conventional specimen.

In addition, Foraboschi [31] presented an investigation on the prediction of the lifetime of concrete members with EBR, focusing on crack growths in the concrete cover due to the shear stresses, which are introduced by the EBR. An analytical method was derived and compared to experimental results.

### 1.6. Long-Term Monitoring Campaigns

In the following, two different long-term monitoring campaigns over an extraordinary long time will be presented. Monitoring of CFRP strips provides indications about their long-term behaviour, and, consequently, confidence in the use of this material for strengthening civil structures.

Firstly, in a long-term laboratory test that has been ongoing at Empa since 1970, a concrete beam has been strengthened in flexure using a steel plate. A two-component epoxy adhesive was used. The beam was and is still loaded to 87% of the mean ultimate load and is still in a good state. Secondly, a monitoring campaign on a road bridge at the boarder of Switzerland with epoxy bonded CFRP strips has been going on since 1996. Displacements are measured manually by using a mechanical strain gauge.

## 2. Concrete Beam with a Bonded Steel Plate

### 2.1. Introduction

**Figure 11:**
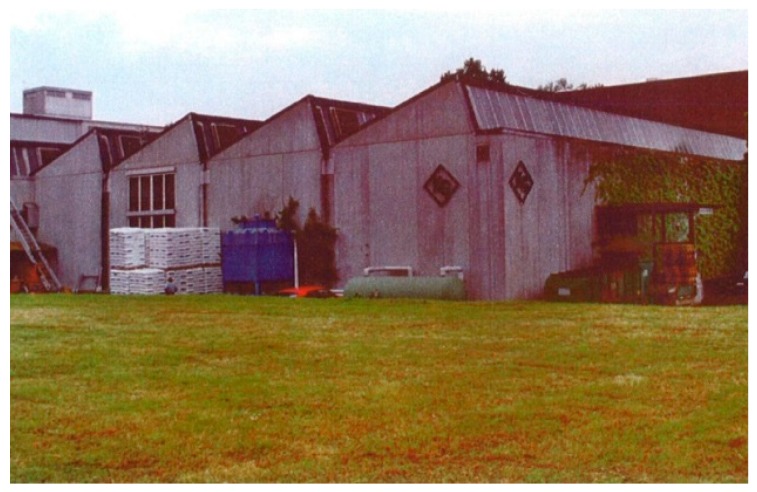
Industrial building in Kreuzlingen in Switzerland, which was strengthened in 1970 by means of steel plates.

**Figure 12:**
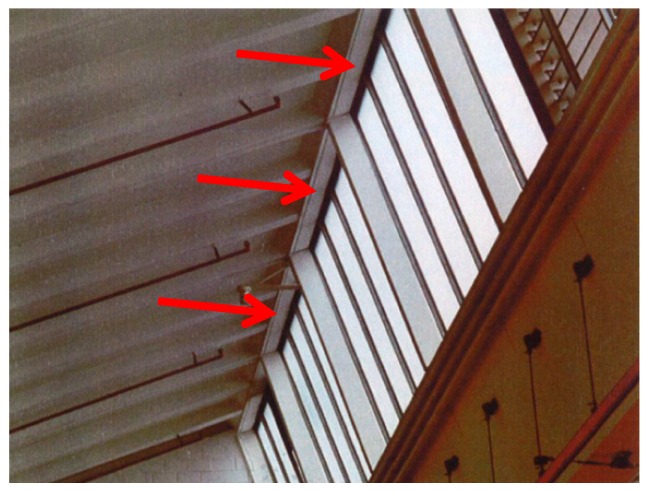
Detail of the inside of the building. The red arrows indicate the location of the steel plates. The photo was taken in the year 1992.

Prefabricated reinforced concrete (RC) elements of the roof of an industrial building (Figure 11) had to be strengthened in 1970. The reason for the strengthening was that cracks were observed and it was found that insufficient internal steel reinforcement existed in the prefabricated RC elements. It was decided that the RC elements shall be strengthened with externally bonded steel plates. Figure 12 shows the strengthened industrial building on a photo in the year 1992. The steel plates are indicated with white arrows in Figure 12. They are hardly visible because of the white paint.

As discussed before, in 1970, the knowledge on the strengthening technique of bonding steel plates with epoxy adhesive to concrete was at the beginning. Therefore, in this year, a test program was performed at Empa to investigate this technique. Several prefabricated RC elements strengthened by epoxy bonded steel plates were tested statically in a four-point bending test, one element was tested under fatigue and one was used for a long-term sustained loading test. This test is still running today and will be presented in this paper. See also [32,33].

### 2.2. Materials and Test Set-Up

The RC elements for the experiments were produced from a Swiss prefabrication company (Element AG, Veltheim, Switzerland) and were delivered to Empa. Unfortunately, only limited documents from the investigation today exist. However, the material properties were reconstructed in the year 2002: the concrete compressive strength after 28 days was f_c,cube,28_ ≈ 28 MPa; in 2002, the concrete strength was f_c,cube_ ≈ 58 MPa, the steel plate is likely to be an ordinary construction steel S235 with a yielding strength of fy ≈ 236 MPa, and the adhesive is a two-component epoxy adhesive probably from the company Ciba-Geigy (Basel, Switzerland) [34].

**Figure 13:**
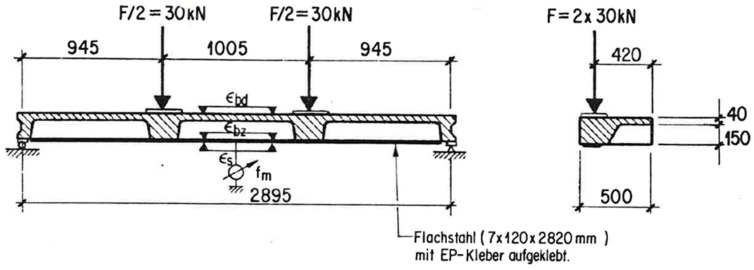
Dimension and measurements of the long-term beam test.

**Figure 14:**
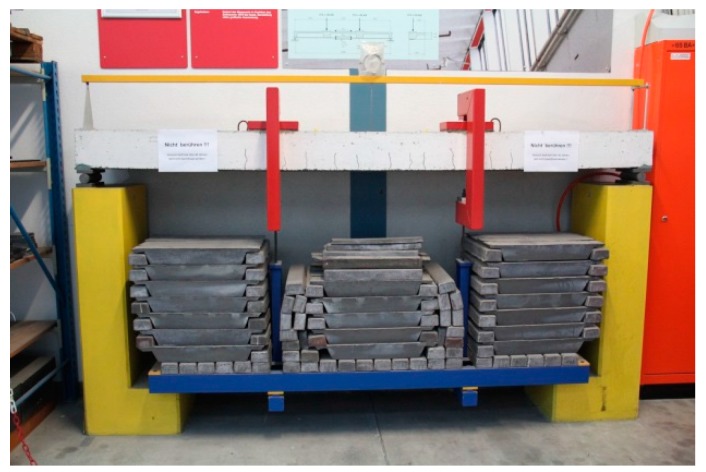
Long-term test at Empa: RC beam strengthened with a bonded steel plate under a sustained load.

**Figure 15:**
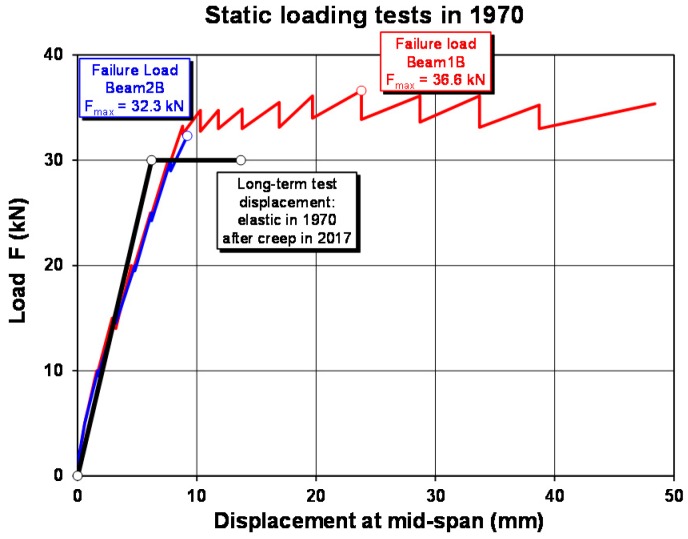
Load-displacement diagram of the static loading tests.

**Figure 16:**
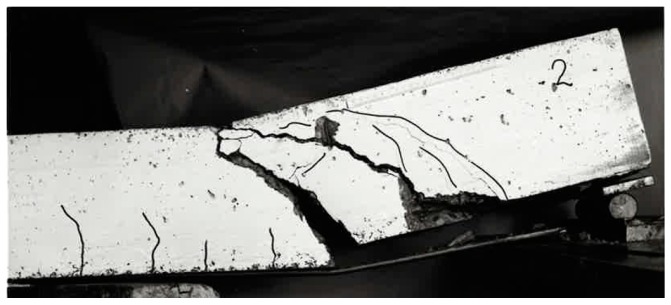
Failure mode in the static loading test on Beam 2B.

The beam is loaded as a four-point bending test (Figure 13 and Figure 14) and constantly loaded with 2 × 30 kN with lead weights. The beam is an edge beam as visible in Figure 12, which explains the nose cross beams visible in Figure 13. The constant load of 2 × 30 kN corresponds to 87% of the mean value of the failure load of the two static tests (Figure 15). This can be considered as a very high value close to failure. A photo of the failure mode during the static loading test of one of the beams is given in Figure 16.

The mid-span displacement of the long-term test is measured with a dial gauge (Figure 14) and the strains with a mechanical strain gauge. In the year 1986, the test had to be relocated in the laboratory and, on this occasion, the loading with concrete blocks was changed to lead weights. Unfortunately, during the reconstruction of the Empa laboratory in 2000, the base for the displacement measurements was damaged. Therefore, these measurements were corrected; however, the strain measurements were not affected from this issue.

### 2.3. Results

**Figure 17:**
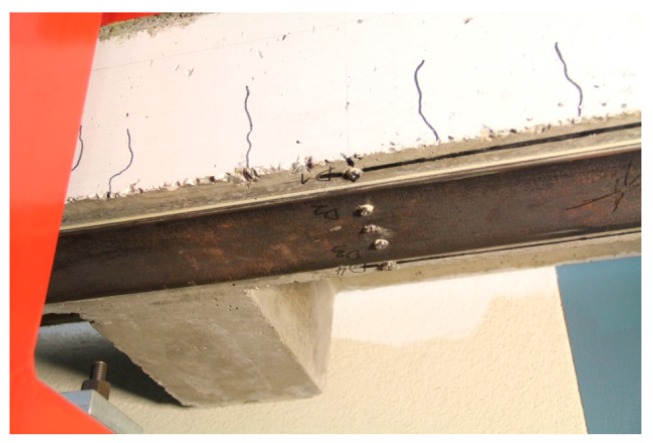
Bottom side of the beam in the year 2015. Surface corrosion is visible on the steel plate.

**Figure 18:**
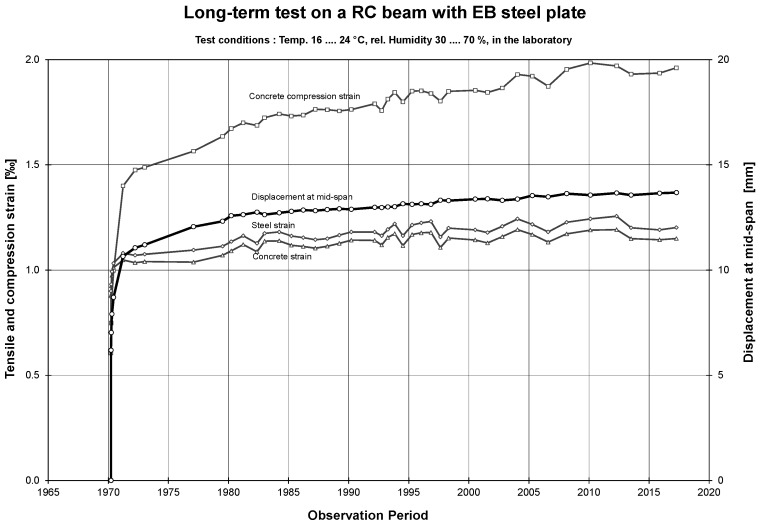
Long-term measurements since 1970 on the RC beam strengthened with an epoxy bonded steel plate.

After 47 years, the beam is still in a good condition. The steel plate has some corrosion at the surface, which is considered as not relevant (Figure 17). The strain-time and displacement-time diagram is given in Figure 18. It shows that the main creep occurs in the concrete in the compression zone. The corresponding creep factors (calculated by the ratio of displacement or strain at 11 April 2017 to 24 March 1970) are given in Table 1. It can be concluded that almost no creep takes place in the epoxy adhesive. 

**Figure 19:**
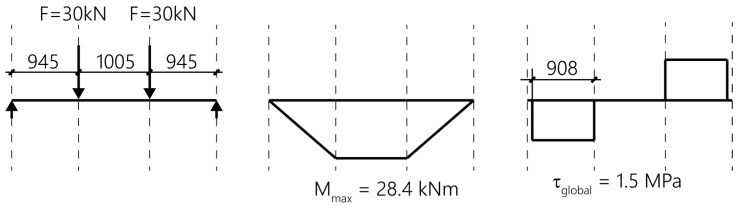
Static system, flexural moment and global shear stress between the steel plate and the concrete surface of the long-term beam test shown in Figure 13.

The tensile stress in the steel plate can be estimated from the measured steel strain of 0.9% by using an elastic modulus of 210 GPa as 189 MPa. The corresponding tensile force is therefore 159 kN (cross-section of steel plate is 7 × 120 mm^2^, Figure 13). A simple check with a comparison of the flexural moments due to loading and resisting results in this force not being correct (assuming no internal reinforcement):

Existing maximum flexural moment from loading: 30 × 0.945 = 28.4 kNm (Figure 19),
Flexural resisting flexural moment: 159 × 0.95 × 0.19 = 28.7 kNm.

If it is assumed that the global shear stress between the loading point and the end of the steel plate is constant, a global shear stress of 1.5 MPa (bond area: 120 × 908 mm, Figure 19) can be determined, which can be considered as a very high stress that was constantly existing over all the years in the beam. However, this study shows that the high loading over 47 years did not influence the externally bonded steel strips negatively.

## 3. Rhine Bridge Oberriet with Externally Bonded CFRP Strips

As already mentioned above, the Ibachbridge near Lucerne in Switzerland was successfully strengthened with CFRP strips in 1991 as a world premiere. These CFRP strips have been monitored only once, in September 2008, due to very difficult access. It was found, as discussed before, that the CFRP strips were still in very good conditions (Figure 9).

**Figure 20:**
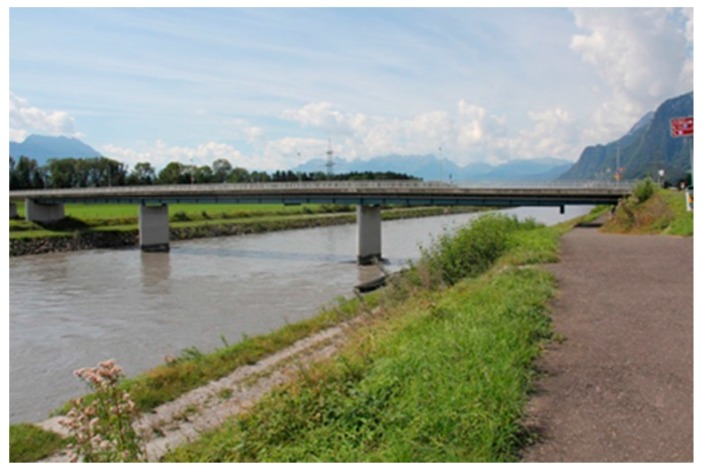
Bridge over the river Rhine near Oberriet in Switzerland.

**Figure 21:**
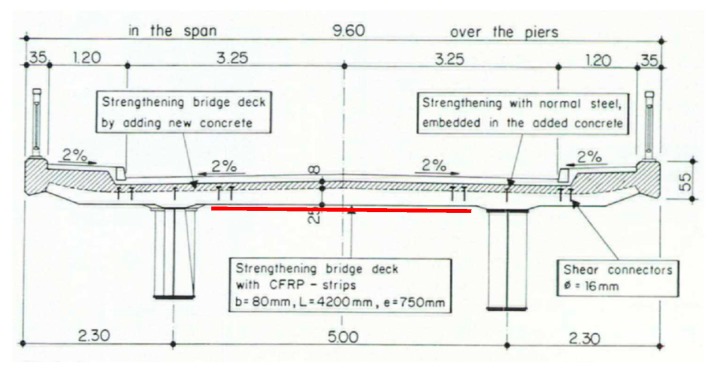
Cross-section of the bridge. Figure taken from [35]. The location of the CFRP strips are indicated with a red line.

In the following, another project that was performed only some years later is described and, due to the easy access, a monitoring campaign was possible. The Rhine Bridge, built in the year 1963, connects Oberriet in Switzerland and Meiningen in Austria and crosses the Rhine River with three continuous spans (Figure 20). The steel-concrete composite superstructure is comprised of two steel girders and a cast-in-place concrete deck (Figure 21). The owner of the bridge is the Canton St. Gallen from Switzerland and the Land Vorarlberg from Austria. 

**Figure 22:**
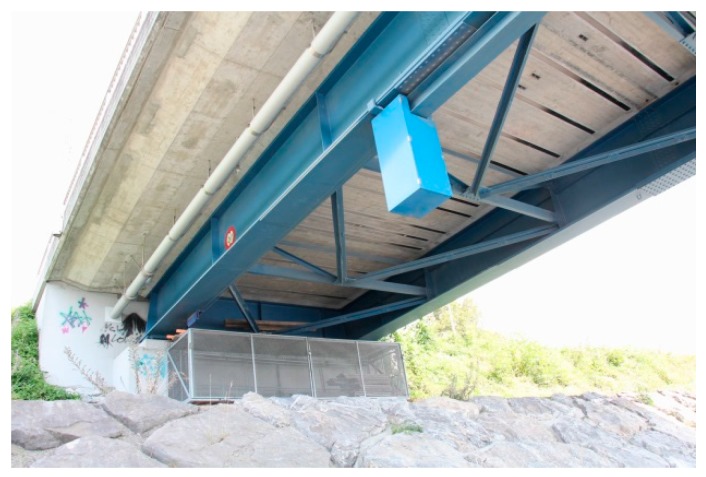
Underside of the bridge. The CFRP strips for the strengthening of the cross-direction are visible between the steel girders.

The strengthening project in 1996 was necessary because of higher road loads and chloride contaminated concrete. On top of the concrete slab, a concrete layer was replaced and a new steel reinforcement for the negative bending moment in bridge cross direction was embedded. On the bottom of the slab, CFRP strips from the Company Sika (Zürich, Switzerland) with a cross-section of 80 × 1.2 mm and a length of 4.2 m were applied in order to strengthen the positive bending moment in the bridge cross-direction (Figure 21 and Figure 22). In total, approximately 670 m strips were used. A description of the design of the strengthening project can be found in Walser et al. [35]. The project engineer was Bänziger & Köppel & Partner from Buchs in Switzerland.

**Figure 23:**
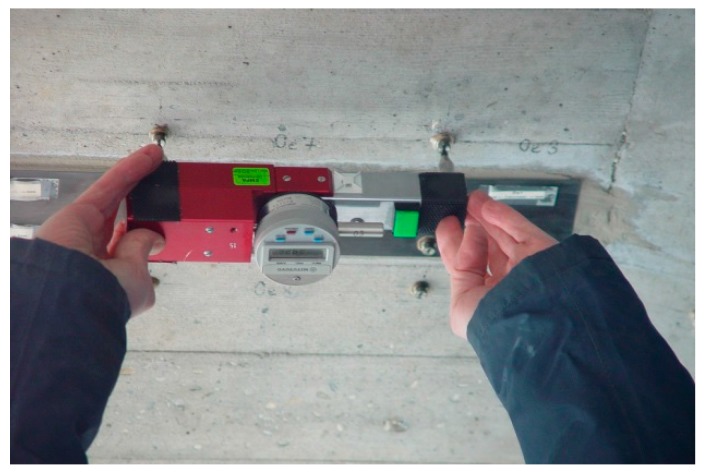
Mechanical strain gauge, which is used for the measurements.

**Figure 24:**
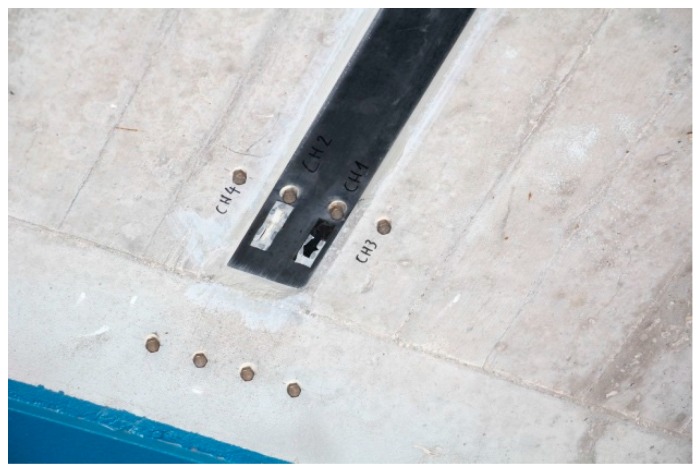
Measurement points at the end of a CFRP strip.

**Figure 25:**
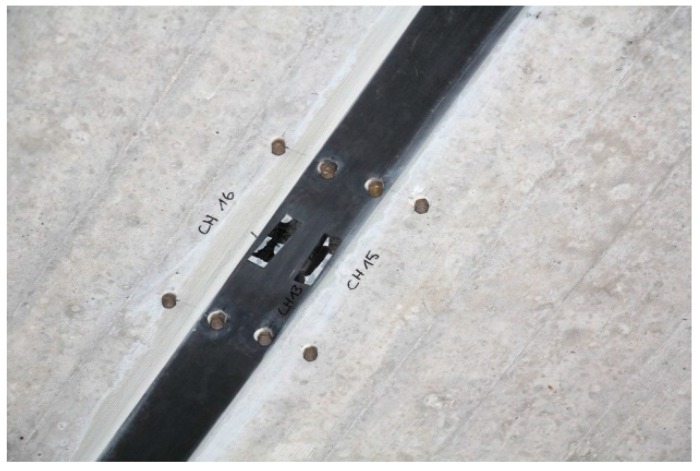
Measurement points at the mid-length of a CFRP strip.

Displacement measurements with a mechanical strain gauge (Figure 23) on two of the CFRP strips and the adjacent concrete has been performed on a regular basis since 1996. The stainless steel measurement points are protected with a threaded cover (Figure 24 and Figure 25). The measurement length of the mechanical strain gauge is 200 mm. Measurements are performed on the CFRP strip at mid-length and strip-end, and at adjacent locations on the concrete. See also [36].

**Figure 26:**
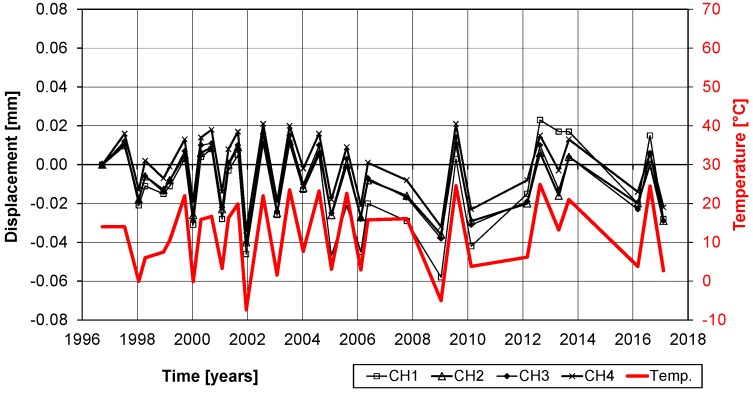
Displacement measurements over the last 20 years. Measurements CH 1 and 2 are from the strip end to the concrete surface and CH 3 and 4 on the concrete surface (see Figure 24).

**Figure 27:**
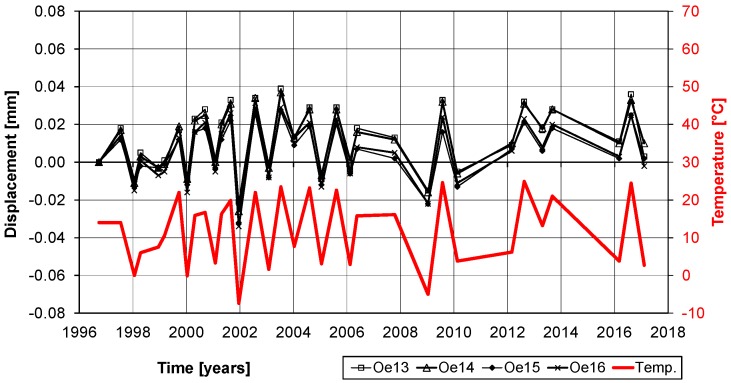
Displacement measurements over the last 20 years. Measurements Oe 13 and 14 are on the strip (at mid-length of strip) and CH 15 and 16 on the concrete surface similar as can be seen on Figure 25.

Some measurements of the variations of the displacements are given in Figure 26 and Figure 27. It is noticeable that the displacements have a strong relation to the temperatures. With a temperature change between winter and summer of ΔT ≈ 25 °C and a temperature expansion coefficient of α_T_ = 10^−5^, a displacement variation of ΔT × α_T_ × L ≈ 0.05 mm has to be expected. Therefore, the measurements in Figure 26 and Figure 27 show only structural expansions and contractions due to seasonal temperature variations and no damage of the bond. Furthermore, the photos in Figure 24 and Figure 25 and visual inspection show that the CFRP strips are still in a good condition. The CFRP strips are bonded at the bottom of the bridge and therefore protected from UV radiation and extensive rain exposure. However, air humidity in foggy weather situations and from the river Rhine, which flows under the bridge, are still expected. Nevertheless, this study shows that all of these effects over more than 20 years did not influence the externally bonded CFRP strips negatively.

## 4. Recommendations

Due to the fact that the steel plates are susceptible for corrosion, the authors recommend using FRPs instead of steel for new EBR strengthening projects. The examples described in this paper prove that FRPs and polymers are very reliable construction materials, if correctly applied (see also [37]). The following measures for durable constructions with FRPs are recommended:

- No water wetting,
- FRPs should be used only for Exposition Classes X0, XC1 (dry) and XC3,
- For other exposition classes: special measures such as coatings are necessary,
- Moderate sustained loading (prestressing, load),
- Service temperatures ≤ 50 °C (ca. 10° lower as *T*_g_)
- If FRPs are exposed to direct UV, there will be a gradual degradation of the polymer matrix at the surface. From a structural point of view this is no problem but it looks ugly. Therefore, in such circumstances, the application of a gelcoat is recommended.

## 5. Conclusions

In the first part of the paper, the impressive history of the development and introduction of the EBR strengthening technique to the construction industry is described. In the 1960s and 1970s, bonded steel plates were used to strengthen reinforced concrete. Then, in the 1980s, the FRPs were introduced and the first site applications were in the 1990s. 

In the second part of the paper, two extremely long-term monitoring campaigns are presented. The following conclusion from these investigations can be made. The 47-year-old test on a beam with an epoxy bonded steel plate and a high sustained load showed that:

- although very highly loaded, the strengthening of an RC beam with externally bonded steel plates is, after 47 years of sustained loading in the laboratory, still in good condition, and no failure was observed,
- the increase of the displacements happens mainly due to creep of concrete,
- the epoxy adhesive for the bonding of the steel plate to the concrete is still in good condition.

The 20-year-old CFRP strips glued on the bottom side of the Rhine Bridge in Oberriet showed that:

- the CFRP strips are still in good condition,
- the measurements show only seasonal temperature deformations.

Lastly, recommendations on how FRPs are correctly applied are given, with the aim to get durable strengthening solutions.

## Figures and Tables

**Table 1:** Overview of the measured displacements and strains shortly after loading and after 47 years of long-term monitoring. Furthermore, the creep factors are given.

Date	Years	Displacement [mm]	Concrete compression strain [‰]	Steel tensile strain [‰]
24 March 1970	0	6.2	0.61	0.90
11 April 2017	47.1	13.7	1.96	1.20
Creep factor:		2.2	3.2	1.3
